# On the Efficiency of Laser Alloying of Grey Cast Iron with Tungsten and Silicon Carbides

**DOI:** 10.3390/ma16186230

**Published:** 2023-09-15

**Authors:** Eugene Feldshtein, Oleg Devojno, Justyna Patalas-Maliszewska, Marharyta Kardapolava, Iryna Kasiakova

**Affiliations:** 1 Institute of Mechanical Engineering, University of Zielona Góra, Prof. Z. Szafrana 4, 65-516 Zielona Góra, Poland; e.feldsztein@iim.uz.zgora.pl; 2 Faculty of Mechanical Engineering, Belarusian National Technical University, Khmelnitsky Str., 9, Build. 6, 220013 Minsk, Belarus; devoino-o@mail.ru (O.D.); margokardo@tut.by (M.K.); i.kosyakova88@gmail.com (I.K.)

**Keywords:** grey cast iron, laser alloying, carbide particulates, microstructure, microhardness

## Abstract

Cast iron is widely used in engineering production and in the surface alloying of workpieces, which is exploited to improve the properties of the material. Research on cast iron is still valid and needed for the manufacturing processes throughout the product life cycle. In this study, the gray, cast iron GJL 200 laser processing is described based on surface alloying with WC and SiC particulates. SEM analysis and XRD analysis, as well as microhardness testing and tribological behavior studies, were employed. It was revealed that laser alloying with carbide particulates affects structural, mechanical, and operational properties compared to cast iron in its initial state. Most importantly, the right choice of laser processing conditions can increase the wear resistance of the cast iron base. The wear resistance after WC alloying was 4–24 times higher compared to the initial material, while after SiC alloying, it was 2–18 times lower than that of the initial material.

## 1. Introduction

Cast iron is one of the most widely used engineering materials and is found in serial and mass production. Both universal and special cast iron, with high wear resistance, are widespread; however, special cast iron is distinguished by its higher cost and the need to develop special alloying technologies. In many cases, it is necessary to ensure improved properties not throughout the whole volume but primarily in its surface layers. The different technologies of surface layer hardening and surface alloying of workpieces are used for this purpose.

The laser alloying of surfaces is a well-developed technology nowadays. This process is like surface melting with a laser; however, the required alloying elements are added to the melt to change the chemical composition of the surface layer. During the interaction of the laser beam with the surface of the part, the pre-applied layer and the substrate are melted, with both melts being intensively mixed and a surface alloy with the required properties being formed during solidification.

Creating a surface layer by laser alloying improves the properties of the material, such as its microhardness, its resistance to abrasion, and its resistance to thermal fatigue, more than is the case with materials treated only by conventional heat treatment.

Zaleski and Skoczylas [1] investigated the influence of shot peening on surface roughness and on the residual stresses of spherical graphite cast iron parts. After treatment, compressive residual stresses formed in the parts. Silva et al. [2] evaluated the influence of shot peening on wear after dry sliding different irons. After shot peening, the austempered samples had an increase in surface hardness but also a decrease in wear resistance. Gu et al. [3] investigated the laser cavitation peening of HT200 gray cast iron. After treatment, the surface roughness increased; however, the presence of a covering layer reduced the surface roughness. Optimization of laser energy for the required surface layer parameters was carried out. Moreover, Yin et al. [4] studied the energy density of the laser spot.

Chen et al. [5] investigated the fatigue wear resistance of gray iron after laser surface modification. After remelting, a surface layer consisted of soft and hard phases. Paczkowska [6] proposed a scheme for laser treatment that combined remelting, alloying, hardening from the solid state, and hardening of the surface layer of gray cast iron, depending on the power density levels and exposure time of the laser beam. Pagano et al. [7] used the laser modification of GJS400-12 cast iron in order to enhance tribological properties. Laser treatment of the surface caused a considerable increase in wear resistance because of the growth of surface hardness. The coefficient of friction has increased due to both adhesive and abrasive processes. Manoj et al. [8] investigated the effectiveness of laser cladding of powders based on AISI 4140 steel, SS420 steel, and the cobalt alloy Stellite 6. Gray cast iron was used as the substrate. Samples based on AISI 4140 and Stellite6 provided a stable coefficient of friction, in contrast to samples from SS420. Surfacing increased the wear resistance of the friction layer in the direction of AISI 4140, SS420, and Stellite alloys. Ceschini et al. [9] studied the influence of laser treatment of GJS400 and GJL300 cast iron on the behavior of both the microstructure and the dry sliding wear. Laser processing increased the wear resistance of cast iron while increasing the coefficient of friction. Wang et al. [10] studied quench-tempering and laser-hardening effects on the wear behavior of gray cast iron. It has been found that laser hardening formed a hardened zone containing ledeburite and a HAZ (heat-affected zone) containing martensite, which were of different hardnesses. The quench-tempering processing caused higher wear resistance in gray cast iron compared to the initial state but lower wear resistance in austempered gray cast iron compared to the initial state. Feng et al. [11] studied the microstructure and mechanical properties of a high-chromium cast iron after laser quenching and laser shock peening and found the character of structural-phase changes, residual stresses at the hardening zone and transition zone, dislocation density, and microhardness. Friction coefficients and wear rates have been significantly reduced. Chen et al. [12] compared the features of high-chromium cast iron after conventional hardening and laser hardening. Based on the analysis of changes in microhardness, microstructure, residual austenite content, grain sizes, content of various phases, and tribological behavior, the advantages of laser hardening were shown. Thilipkumar et al. [13] investigated the tungsten surface modification of Ni-Hard 4 cast iron using a gas tungsten arc source and determined that the microstructure, the wear rate, and the hardness of the alloyed iron improved significantly. Feldshtein et al. [14] analyzed changes in the microstructure, microhardness of the surface layer, and wear resistance of gray cast iron after laser hardening. With the right choice of surface energy density, stable and low friction coefficients and a five- to tenfold increase in the wear resistance of gray cast iron were provided compared to the initial state.

Yang et al. [15] examined the effect of laser alloying by Cr and Ni on the thermal fatigue resistance of cast iron and found that the nano-hardness of the alloying zone increased significantly due to the formation of a homogeneous microstructure which upgraded the chemical composition and refined grains. Yilbas et al. [16] performed laser alloying of a cast iron surface in order to control resistance to surface corrosion. Before laser treatment, a film was formed on the surface of the workpiece containing 15% of SiC particles and 85% of carbon. As a result, a dense layer consisting of fine-grained, partially dissolved SiC was formed, which increased the corrosion resistance of the surface. Selech et al. [17] compared two types of cast iron surface treatments, namely thermal spraying with eutalloy 10,112 and laser alloying with SiN, in terms of their abrasive wear rate. Laser alloying increased hardness by 2.5 times and thermal spraying by 3 times. Wear to the parts after laser alloying was five times less and after thermal spraying six times less than the wear of the parts in their original state. Szymański et al. [18] examined the effect of a TiC/Fe coating formed by self-propagating high-temperature synthesis on gray cast iron and low-carbon steels. The effectiveness of the coating was shown in terms of microhardness, microstructure, and wear rate. Zhang et al. [19] studied the properties of nodular cast iron after pre-spraying a (Al_2_O_3_ + Fe_3_O_4_ + SiO_2_) layer and subsequent remelting by fiber laser under optimal laser power and scan speed. It was shown that the morphology, structure, and operational characteristics of the laser-remelted surface have undergone significant positive changes. Yilbas et al. [20] investigated the laser alloying of dual matrix cast iron by introducing WC particles. The laser treated layer consisted of a dense area composed of fine grains and WC particles, with dendritic and feather-like structures below the dense area and the HAZ. Janicki et al. [21] studied the formation of TiC particles in an iron matrix during the laser alloying of a ductile iron surface. The morphology and the volume of TiC particles depended on the amount of Ti in the molten pool, which, in turn, strongly depends on the density of the laser energy. Paczkowska and Selech [22] evaluated the efficiency of laser alloying with B and a mixture of B and Cr of the surface layer of the cast iron parts. Compared to the surface layer of the basic material -white cast iron-, after laser alloying, a more homogeneous and additionally strengthened microstructure was formed with an increase in hardness by about two times in the case of B and by three times for the mixture of B and Cr. Wear tests showed that this improved the wear resistance of the machined parts more than 2-fold. Xiao et al. [23] studied the phase composition, microstructure, microhardness, and tribological behavior of laser cladding Fe-WC coatings, as well as the effect of the thermal damage of WC particles on the evolution of the structure and the wear resistance. The main mechanism of wear in iron-based coatings, containing WC particulates, was abrasive wear, accompanied by adhesive wear and third body formation. Janicki [24] analyzed TiC-reinforced layers obtained on ductile iron by laser surface alloying and showed that the volume of TiC particles significantly affects the morphology and mechanical properties of ductile iron. Increasing the TiC content decreases both the wear rate and the friction coefficient.

Thus, the laser surface alloying of cast iron parts demonstrated itself to be highly efficient. Data on changes in their basic material characteristics has been obtained; however, the operational (tribological) characteristics are limited to dry abrasive friction conditions only. The aim of this research was a complex analysis of the efficiency of the laser alloying of gray cast iron, in particular regarding differences in the surface layer microstructure and microhardness as well as the surface layer wear resistance under friction with oil lubrication.

## 2. Materials and Methods

### 2.1. Laser Unit and Conditions of Laser Alloying

**Figure 1:**
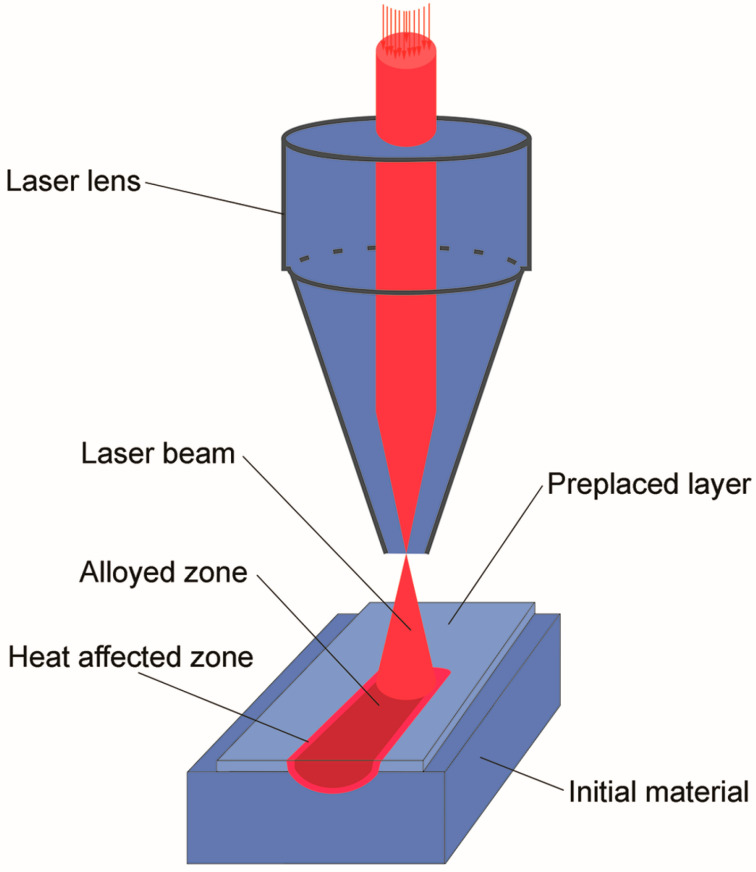
The scheme of laser alloying with preplaced non-adherent coating.

The laser unit “Kometa” of “Istok” NPO with a wavelength of 10.6 nm and power of 1.2 kW was used for coating processing. It was a continuous-action CO_2_ gas laser that is intended primarily for processing hard materials and materials of considerable thickness. To evaluate the influence of the laser treatment on changes in the surface layer properties, alloying followed by melting was investigated (Figure 1).

Laser processing was carried out with laser beam diameters of 1 and 2 mm and melting speeds of 100 and 600 mm/min. The overlapping ratio was 1.0. The mutual effect of laser beam diameter and melting speed was evaluated using a 2^2^ full factorial design, whose matrix is shown in Table 1. To simplify the analysis of the impact of alloying conditions on the surface layer properties of the hardened cast iron, one of the main parameters of the laser processing, namely the surface energy density, which corresponded to the investigated combinations of the laser beam diameter and speed, was additionally taken into account.

A paste-like composition of alloying carbides with BF-6 glues and an acetone solution was used for precoating. After drying, its thickness was 0.1 ± 0.02 mm. When laser alloying, an add-in mixture of 75 vol.% carbide particulates with water was hand-brushed on the sample surface. After drying, its thickness was 0.1 ± 0.05 mm. The statistical analysis was completed using Statistica 13 software. To validate the results, the tests were performed three times at each measuring point.

### 2.2. Samples Preparing

The samples for microstructural studies were ground using an abrasive paper. The rough grinding was realized with Nr 22 or Nr 16 abrasive papers, and the finish grinding with Nr 4 or Nr 3 abrasive papers (the symbols according to ISO standards). The SiC grains were used as abrasives. Then, polishing was carried out using a rotating wheel covered with a cotton felt under water, and a powder mixture of Cr_2_O_3_ and Al_2_O_3_ oxides of 3–5 μm size for 3–4 min, until the last risks left on the surface were removed. To reveal the structure of the metal after polishing, the samples were etched with a 4% solution of HNO_3_ acid in ethyl alcohol.

### 2.3. Tribological Testing

Friction and wear features were tested under concentrated contact conditions, and a “roller-block” scheme was used. Samples (blocks) were made after the laser alloying of iron and tested. Counter-bodies (rollers) were made of AISI 1045 steel. The roller’s hardness was 45–51HRC. Samples were machined by an electro-corundum wheel under constant machining conditions. Loads of 500 N and 1000 N were applied with a 0.45 m/s sliding speed. The stand-alone test time was 1 h. LAN-68 machine oil type was used as a lubrication medium (Jaśle refinery, Jasło, Poland). The oil drop-feed method was applied to lubricate the friction zone, and the droplet flow was 30 drops per minute. When testing, the momentary COF and temperature T were registered over time. The volumetric wear of the sample tested *V_w_* and wear rate *I_V_* were specified based on the width of wear traces measured. For calculating the following equations, momentary COF:

(1) $$f=\frac{2M}{FD_{r}}$$

where: *M*—the moment of friction; *F*—the load; *D*_r_—the roller diameter, mm.

Volumetric wear *V*_w_:

(2) $$V_{w}=\frac{D^{2}B}{8}\left\{2\arcsin\left(\frac{s}{D_{r}}\right)-\sin\left[2\arcsin\left(\frac{s}{D_{r}}\right)\right]\right\}$$

where: *B*—the width of the sample; *s*—the average width of the wear groove.

Wear rate *I_V_*:

(3) $$I_{V}=\frac{V_{w}}{L}$$

where: *L*—the friction path.

### 2.4. Measuring Equipment

The thickness of the preplaced WC or SiC layer was controlled using the MiniTest 7400 FH (Elektro Physik, Köln, Germany). A Buehler Micromet 2tester (Spectrographic Ltd., Leeds, UK) was used to measure the surface layer microhardness; a sinker of 100 g was employed. The surface roughness parameters were measured with a TR-200 tester (Salu Tron GmbH, Frechen, Germany). Microstructure features were studied with an MBA microscope (BelOMO Holding, Minsk, Belarus). Worn surfaces were analyzed using a JSM-5600LV (JEOL Ltd., Tokyo, Japan) scanning electron microscope. The diffractometric analysis was fulfilled with the DRON-3 unit (Bourevestnik JCS, Saint-Petersburg, Russia) and the Cu-Κ_α_ emission. Tribological tests were performed using the friction tester of the A-135 type. The friction moment and time of friction were registered by suitable sensors, and the temperature in the friction zone was controlled by the chromel-alumel thermocouple. The wear of samples was controlled by a Dino-Lite digital microscope (AnMo Electronics Corporation, New Taipei City, Taiwan), and the measurement accuracy was 0.001 mm.

## 3. Results and Discussion

### 3.1. The Start Material

**Figure 2:**
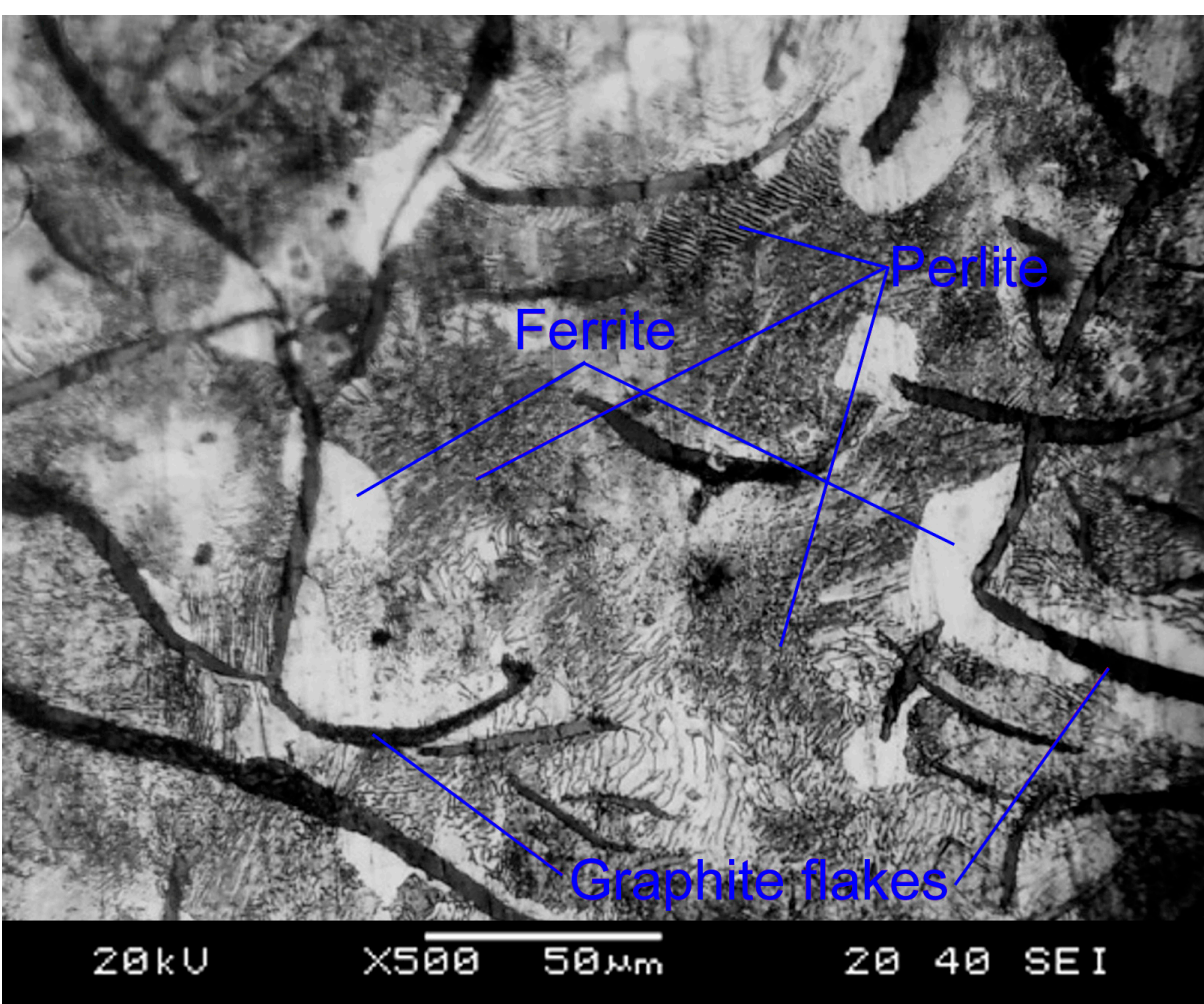
Microstructure of the based material.

The gray cast iron EN-GJL 200 was tested as the start material (Figure 2). It has a pearlitic-ferritic microstructure and contains 3.10−3.40% C, 1.90−2.30% Si, 0.60−0.90% Mn, ≤0.15% P, and ≤0.15% S (EN 1561:1997). It is characterized by a Brinell hardness of 170−210a tensile strength of 200 MPa, a compressive strength of 800 MPa, and a shear stress of 230 MPa.

### 3.2. Particulates to Laser Alloying

**Figure 3:**
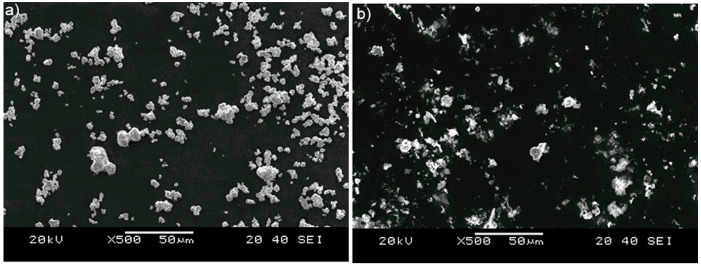
WC (**a**) and SiC (**b**) particulates.

Hard ceramic particulates of tungsten carbide WC with a size of 2 to 20 μm and silicon carbide SiC with a size of 2 to 10 μm were used for surface layer alloying (Figure 3).

### 3.3. The Surface Roughness Changes

It was revealed that the microroughness profiles after samples were ground in the initial state and after laser alloying differed. In the case of laser alloying, the surface is more uniform, with a smaller height of microroughness. Technological parameters of laser alloying affect the roughness parameters insignificantly, which does not take into account the influence of friction surfaces’ microroughness on their tribological characteristics.

### 3.4. The Microstructure of the Surface Layer of GJL 200 Gray Cast Iron after Laser Alloying with Tungsten Carbides

**Figure 4:**
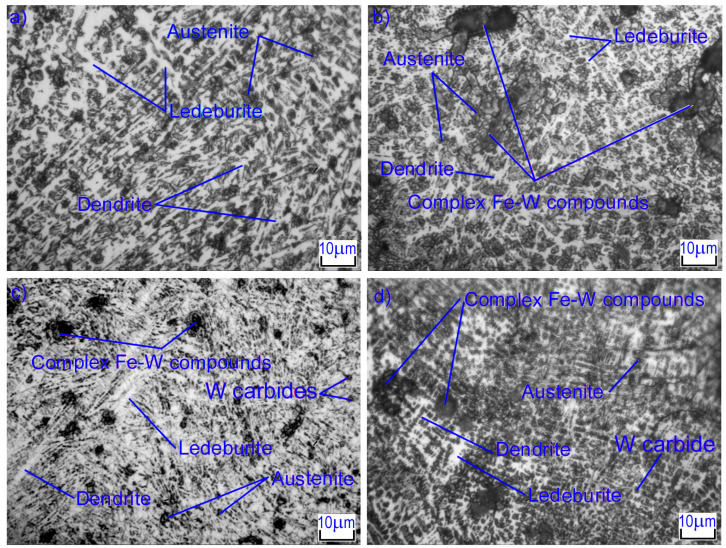
Microstructures of strengthened layers after laser alloying with tungsten carbides: (**a**) *d* = 1 mm, *V* = 100 mm/min, (**b**) *d* = 2 mm, *V* = 100 mm/min, (**c**) *d* = 1 mm, *V* = 600 mm/min, (**d**) *d* = 2 mm, *V* = 600 mm/min.

Laser alloying of cast iron with tungsten carbides significantly changes the microstructure of the surface layer. The main factors influencing the formation of the laser-treated layer are the high heating rate of the surface layer (in our case, 10^5^–10^7^ K/s) due to the high energy density as well as the high cooling rate due to heat removal into the substrate. High heating rates lead to a significant shift in the points of phase transformations with diffusive characteristics. High cooling rates of the zone molten by laser radiation lead to incomplete diffusion processes, the formation of a large number of embryos of different phases, and thus the formation of a fine-grained, non-equilibrium structure. Figure 4 shows the microstructures of the melting zone of gray cast iron after laser alloying with tungsten carbide. The differences in microstructures, depending on the diameter and speed of the laser beam, can be clearly observed. At low laser beam speeds (*V* = 100 mm/min), volumetric heating of that part occurs where the layer alloyed has been in the molten state for quite a long time, 5–10 s. During this time, the layer of alloy crystallizes and cools down completely. Laser melting results in the formation of a metastable globular structure based on iron-based solid solution grains. The structure is armored by first- and second-order dendrites, consisting of alloyed α-Fe and γ-Fe. A finely dispersed eutectic is formed between the axes of the dendrites. Complex compounds of tungsten with iron are also observed in the structure; this will be discussed below. The diameter of the laser beam is negligible in its impact.

**Figure 5:**
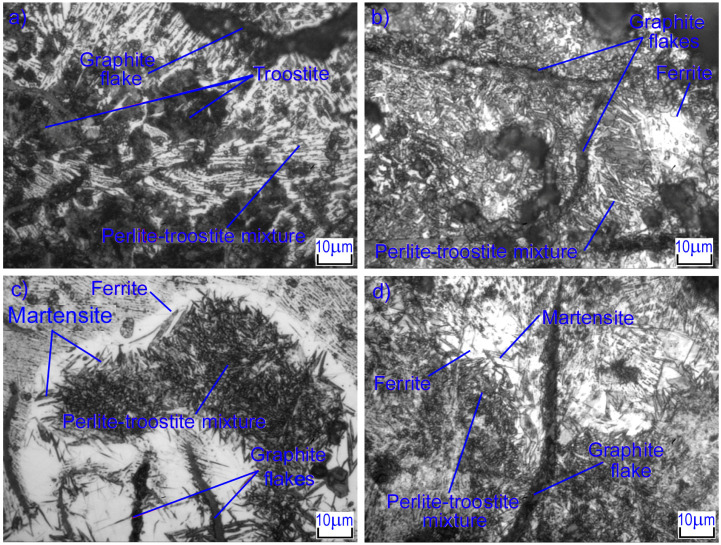
HAZ microstructures after laser alloying with tungsten carbides: (**a**) *d* = 1 mm, *V* = 100 mm/min, (**b**) *d* = 2 mm, *V* = 100 mm/min, (**c**) *d* = 1 mm, *V* = 600 mm/min, (**d**) *d* = 2 mm, *V* = 600 mm/min.

At high laser beam speeds (*V* = 600 mm/min), the melting zone has a structure that is almost completely composed of ledeburite. Graphite inclusions are completely dissolved and are not observed in the melting zone; however, there are significant amounts of WC and W_2_C tungsten carbides. Small dendrites of austenite are observed in the structure; they grow during crystallization and are surrounded by dispersed ledeburite. First- and second-order axes’ dendrites were observed, and less often, third-order axes. In essence, there is simply not enough time for them to form as crystallization ends. Under conditions of the minimal surface energy of the laser beam, the maximum amount of fine iron and tungsten carbides (~31%) is formed; these are located between the branches of the second-order dendrites. The interface between the melted zone and the heat-affected zone (HAZ) is indefinite in character. There is no clear dividing line, and areas of melted zones are embedded in the transition zone in some places. The HAZ is formed during solid-state hardening, is distinguished by a large inhomogeneity of the structure in depth (Figure 5), and is further characterized by an increased rate of heating and cooling compared to the molten zone.

It was observed that during laser processing, the iron matrix around the graphite is melted and saturated with carbon in the upper area of the HAZ. Separate zones of some of the structural components are formed in the HAZ: A ferrite-dominated layer is formed near the graphite, then martensite, and, finally, a pearlite-troostite needle structure. In the lower area of the HAZ, where the saturation of the cast iron matrix with graphite is very low, the structure is martensite and residual austenite. At the boundary with the initial cast iron, the process of authentication of pearlite remains incomplete, and undissolved particles of cementite can be observed here. In this area, lower-hardness pearlite dominates (HV_100_ = 320–350). In the upper part of the HAZ, increasing the laser spot speed decreases the degree of carbon saturation of the matrix around the graphite inclusions. In the lower part of the HAZ, the incompleteness of austenitization during heating increases; therefore, the solid solution is less saturated with carbon. As a result of laser alloying, needle-shaped martensite, and ledeburitic eutectics are formed in the HAZ. Plate-shaped graphite is also observed. The areas of ledeburitic eutectic are a continuation of the melted layer and are rounded in shape. These areas limit the zones in which graphite inclusions were previously located and which had time to dissolve when heated, enriching the austenite with carbon. In addition, areas with the largest graphite inclusions, partially dissolved, are also observed. They are also embedded in martensite and located in places where the temperature and holding time were insufficient to completely dissolve the inclusions. Further studies showed the hardness of needle martensite as HV_100_ = 500–775.

### 3.5. The Microstructure of the Surface Layer of GJL 200 Gray Cast Iron after Laser Alloying with Silicon Carbides

**Figure 6:**
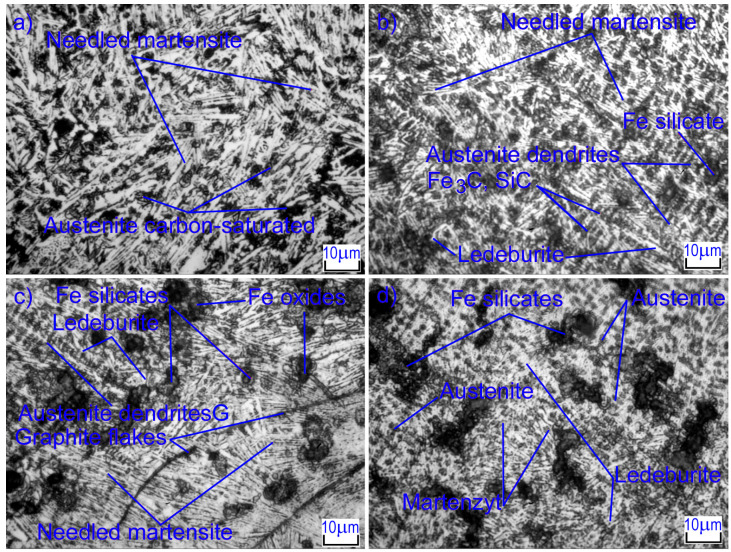
Microstructures of strengthened layers after laser alloying with silicon carbides: (**a**) *d* = 1 mm, *V* = 100 mm/min, (**b**) *d* = 2 mm, *V* = 100 mm/min, (**c**) *d* = 1 mm, *V* = 600 mm/min, (**d**) *d* = 2 mm, *V* = 600 mm/min.

Laser alloying of gray cast iron with silicon carbides also significantly changes the microstructure of the surface layer. The microstructures of the laser-alloyed zone under different conditions are shown in Figure 6. At low laser beam speeds (*V* = 100 mm/min) and high surface energy density, the volumetric heating of the layer takes place where the coating has been in the molten state for quite a long time. In this case, the initial pearlitic structure in the cast iron matrix was transformed into needled martensite and carbon-saturated austenite. The microhardness of needled martensite is HV_100_ = 500–775, which provides high hardness for the alloyed layer.

**Figure 7:**
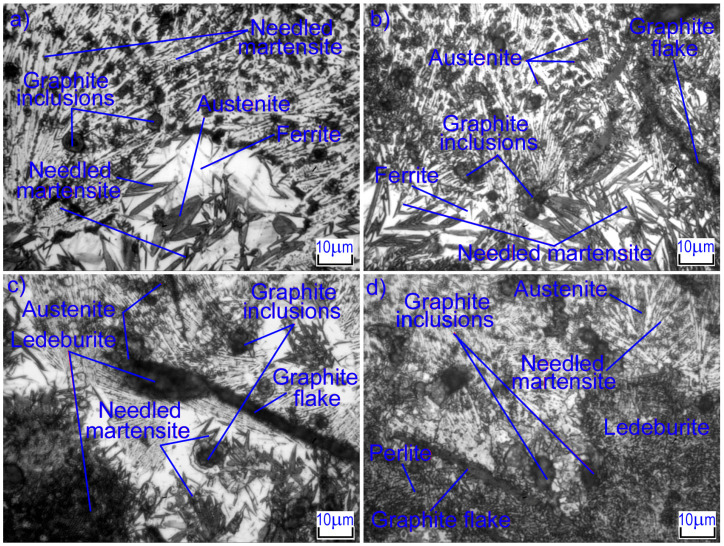
HAZ microstructures after laser alloying with silicon carbides: (**a**) *d* = 1 mm, *V* = 100 mm/min, (**b**) *d* = 2 mm, *V* = 100 mm/min, (**c**) *d* = 1 mm, *V* = 600 mm/min, (**d**) *d* = 2 mm, *V* = 600 mm/min.

As the diameter and speed of the laser beam increase, the energy density in the surface layer decreases. In this case, the microstructure includes small dendrites of austenite, which were growing during the crystallization of the molten metal. Dendrites have predominantly first-order axes, indicating a high rate of crystallization of the alloy. When the laser beam speed is higher, the dendrites are smaller. Inclusions of needle martensite, iron oxides, and silicon and iron carbides, which are located between the branches of the second order of dendrites, are also observed. The interface between the melted zone and HAZ has an implicit character: there is no clear boundary line, and areas of molten areas are embedded in the HAZ in some places (Figure 7).

HAZ is formed under solid-state hardening conditions. It is distinguished by a large inhomogeneity of the structure along the depth and is characterized by an increased rate of heating and cooling as compared to the alloyed zone. As a result of laser processing, needle-shaped martensite, graphite, and areas of ledeburitic eutectic are observed in the HAZ structure. These areas are a continuation of the molten layer and are rounded in shape. They limit the places where graphite inclusions were previously located, since these had time to dissolve during heating, enriching the austenite with carbon. In addition, in the microstructure, there are areas with partially dissolved graphite inclusions, which are the largest of them. These areas are also embedded in martensite and are located in places where the temperature and holding time were insufficient to completely dissolve the inclusions. It was observed that the iron matrix around the graphite inclusions was melted and saturated with carbon in the upper area of the HAZ. Studies revealed that areas of the different structural components are formed into HAZ: A light layer is formed near the graphite, evidently with a ferrite predominance, and then martensite. In the lower area of the HAZ, where the saturation of the cast iron matrix with graphite is very negligible, the structure consists of martensite and residual austenite. Near the boundary with the base metal, the process of pearlite austenization is not complete, with undissolved cementite particles and the predominance of pearlite base and hardness HV_100_ = 320–350 being observed here.

### 3.6. The Phase Composition of the Surface Layer of Gray Cast Iron GJL 200 after Laser Alloying with Tungsten and Silicon Carbides

**Figure 8:**
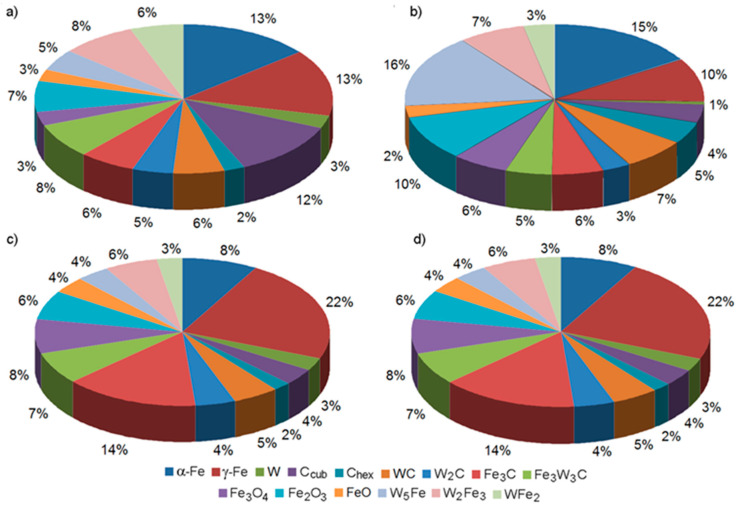
The results of XRD analysis of gray cast iron after laser alloying with tungsten carbides: (**a**) *d* = 1 mm, *V* = 100 mm/min; (**b**) *d* = 2 mm, *V* = 100 mm/min; (**c**) *d* = 1 mm, *V* = 600 mm/min; (**d**) *d* = 2 mm, *V* = 600 mm/mm.

The XRD analysis of cast iron GJL 200 in its initial state registered the presence of α-Fe, C_hex_ graphite, a small amount of C_cub_ carbon with a cubic crystal lattice, and iron carbides Fe_3_C, which are components of pearlite. During the laser remelting of gray cast iron with a layer of pre-applied carbides, a number of complex chemical compounds are formed due to high temperatures and the actively turbulent mixing of the initial materials. Thus, when alloying with tungsten carbide, various iron oxides, various iron-tungsten intermetallides, and complex iron-tungsten carbides are formed (Figure 8). Carbides W_2_C in the amount of 3–5% and a small amount of pure tungsten are also formed; the amount of initial α-Fe is relatively small; however, depending on the conditions of laser alloying, austenite γ-Fe in the amount of 10–22% occurs.

**Figure 9:**
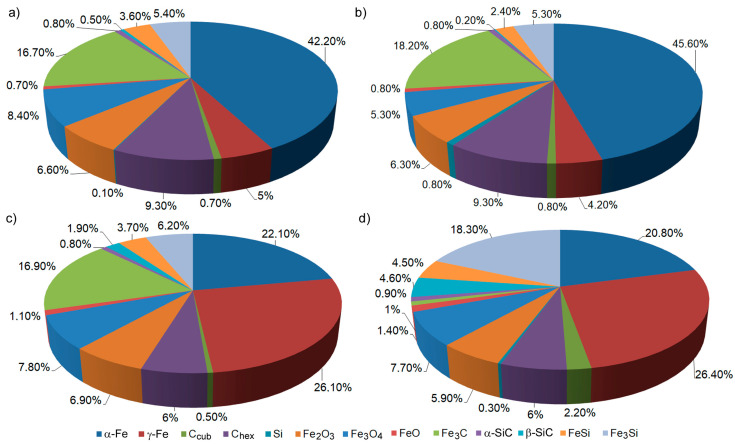
The results of XRD analysis of gray cast iron after laser alloying with silicon carbides: (**a**) *d* = 1 mm, *V* = 100 mm/min; (**b**) *d* = 2 mm, *V* = 100 mm/min; (**c**) *d* = 1 mm, *V* = 600 mm/min; (**d**) *d* = 2 mm, *V* = 600 mm/mm.

When alloying gray cast iron with silicon carbides, various iron oxides, a number of iron silicides, and two different modifications of silicon carbide are formed (Figure 9). The presence of C_hex_ graphite, along with a small amount of C_cub_ carbon with a cubic crystal lattice and iron carbides Fe_3_C, included in pearlite, was registered, as in the case of alloying with tungsten carbides. The amount of initial α-Fe in the alloyed layer decreases, and the amount of austenite γ-Fe increases significantly when the speed of the laser beam is increased. The diameter of the laser beam does not materially affect the change in the content of austenite.

### 3.7. Changes in the Microhardness of the Surface Layer after Laser Alloying

**Figure 10:**
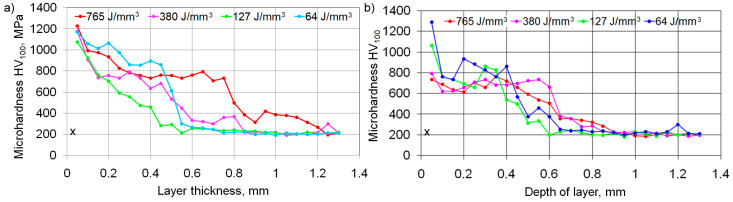
The microhardness distribution along the depth after alloying with WC particulates (**a**) and SiC particulates (**b**) as a function of laser beam energy density (x—an initial cast iron microhardness).

Laser alloying of GJL 200 gray cast iron has a considerable effect on the surface layer microhardness provided it increases (Figure 10). Depending on laser processing parameters, the microhardness after alloying with tungsten carbides increases 3.5–4.5 times, and after alloying with silicon carbides, it increases 3.0–4.0 times, compared with the microhardness HV_100_ = 190–230 of initial cast iron.

**Figure 11:**
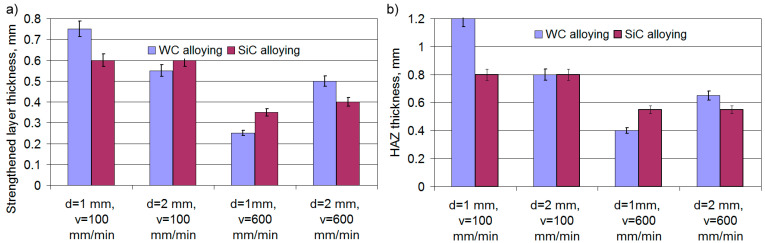
Influence of laser alloying parameters on the strengthened layer thickness and the HAZ thickness after alloying with WC particulates (**a**) and SiC particulates (**b**).

The thickness of the strengthened layer after alloying with tungsten carbides, as well as the HAZ thickness, changes in dependence on laser processing parameters, mostly on the laser beam speed (Figure 11). This is related to the conditions of heat removal and the solidification rate of the molten pool. The diameter of the laser beam has practically no influence on the strengthened layer thickness; it is also safe to say that there is no interaction between the laser processing parameters being studied.

### 3.8. The Tribological Behavior of the Gray Cast Iron Surface Layer after Laser Alloying

**Figure 12:**
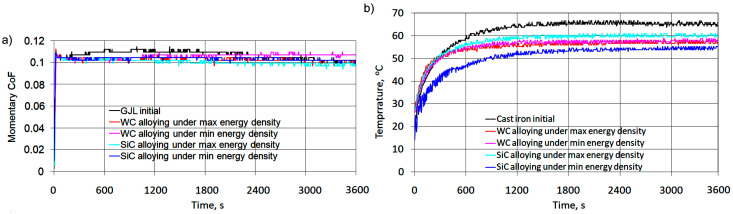
Typical changes in time of momentary CoFs (**a**) and friction temperature (**b**) after laser alloying of cast iron.

Analyzing changes in time of momentary CoFs of cast iron samples after laser alloying, it is possible to reveal that CoFs remain practically constant in the case of applying relatively small loads, i.e., laser processing conditions, alloying material, and friction time have no influence on them (Figure 12a). When the load is increased two-fold, the CoFs of alloyed cast iron remain stable with a slight increase in value compared to smaller loads. However, as compared to the cast iron in the initial state, in this case, the CoFs after laser alloying decrease by ~1.2 times.

The running-in time of the friction surfaces does not exceed 10 min, after which the temperature remains practically constant, regardless of the alloying substance and laser processing parameters (Figure 12b); however, for cast iron in the initial state under conditions of significant loads, it is 1.2 times higher in comparison with materials after laser alloying and reaches ~140 °C.

### 3.9. The Wear Behavior of the Surface Layer of Gray Cast Iron after Laser Alloying

**Figure 13:**
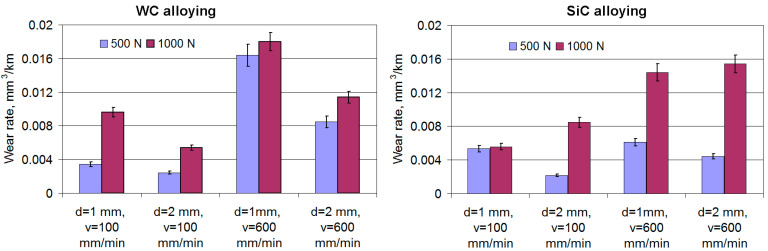
The effect of laser processing conditions on the wear rate of alloyed gray cast iron under a load of 500 N and a load of 1000 N.

In the case of WC alloying, regardless of the load level, the wear rate of the gray cast iron surface layer is minimal when the laser beam speed decreases but the diameter of the laser beam increases, i.e., under the average level of the surface energy density (Figure 13). In the case of TiC alloying and under low loads, the wear rate changes in a similar way. However, with increased loads, the minimum wear rate is registered for the case of processing under the minimum speed and diameter of the laser beam (Figure 13), i.e., under the maximum surface energy density. In all cases, laser beam speed affects the beam more intensively, and there is no interaction between laser beam speed and diameter.

After WC alloying, the selection of rational processing conditions makes it possible to increase the wear resistance of cast iron by 4–24 times compared to the initial material and after SiC alloying by 2–18 times. Such changes are caused by changes in the microhardness and microstructure of the surface layer, which are related to the surface energy density level as well as to the friction conditions and changes in the surface layers. It should be emphasized that, in these studies, the overlap ratio was 1.0. Such an approach allowed changes in the degree of surface layer reinforcement to be made after laser alloying, depending on the position of the laser beam trace, thus positively influencing the running-in and lubrication of friction surface conditions and the intensity of their local wear. SEM studies of the worn surfaces of the samples were performed after the friction cycle under a load of 1000 N, since, with higher loads, differences in wear characteristics are more noticeable.

**Figure 14:**
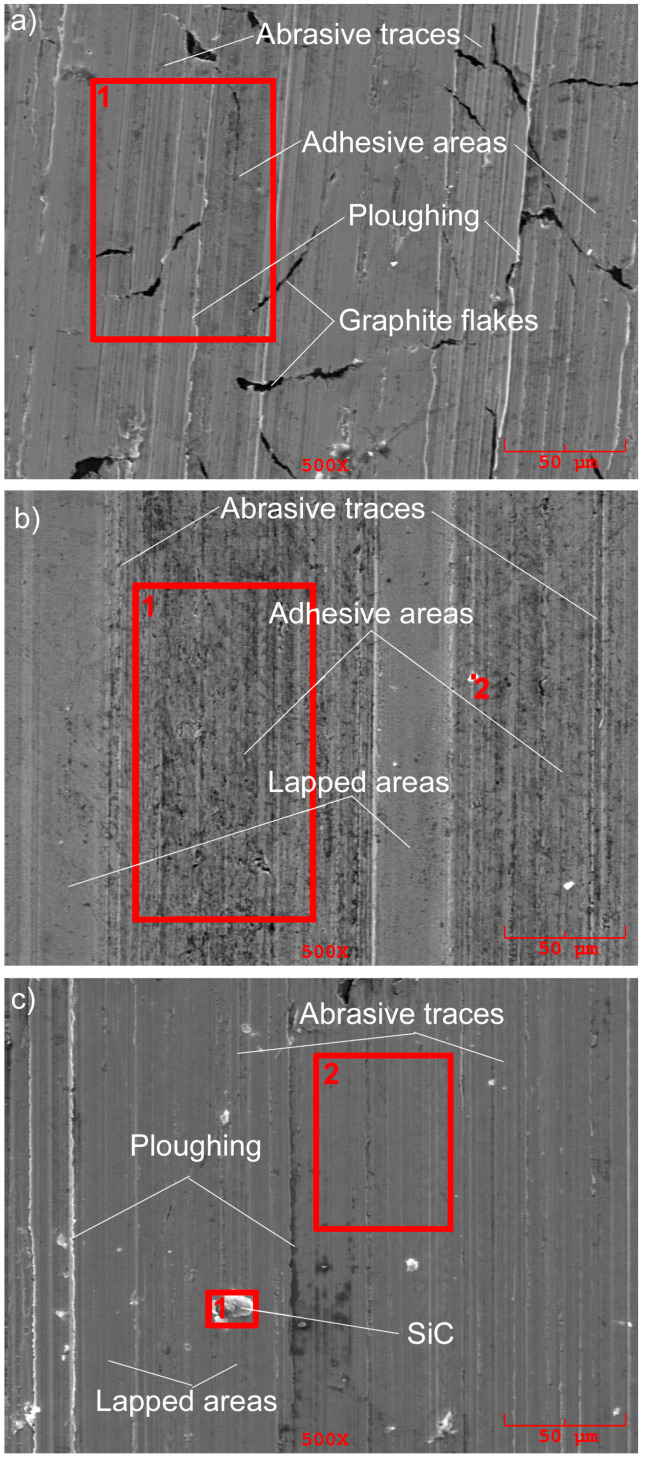
The morphology of the worn surfaces of gray cast iron samples in the initial state (**a**), as well as those alloyed with WC (**b**) and SiC (**c**) carbides.

On the worn surface of gray cast iron samples in their initial state (Figure 14a), traces of abrasion, along with small areas of adhesive seizure, as well as ploughing, can be observed. In the case of WC and SiC alloying (Figure 14b,c), it is possible to distinguish areas where the mutual overlapping of sample materials with the counter-body takes place on the worn surfaces and, as a consequence, the friction coefficient is reduced. Such phenomena are characteristic only of surfaces formed under the influence of high surface energy densities; at lower energy densities, the overlapping areas disappear. Small particulates of WC and SiC carbides dissolve in the cast iron matrix during laser remelting, while larger ones remain in the surface layer, activating abrasive wear and causing ploughing.

The SEM analysis registered the presence of tribofilm on friction surfaces, which reduces and stabilizes friction coefficients and increases wear resistance. In tribofilm, the increased content of sulfur and phosphorus in lubricating oil is observed (Table 2 and Table 3).

Separate particulates of WC and SiC carbides, as well as atoms of phosphorus and sulfur presented in lubricating oil, have been registered in the tribofilm formed (Table 4 and Table 5). Graphite, which is present in the initial material, also passes into the tribofilm.

**Figure 15:**
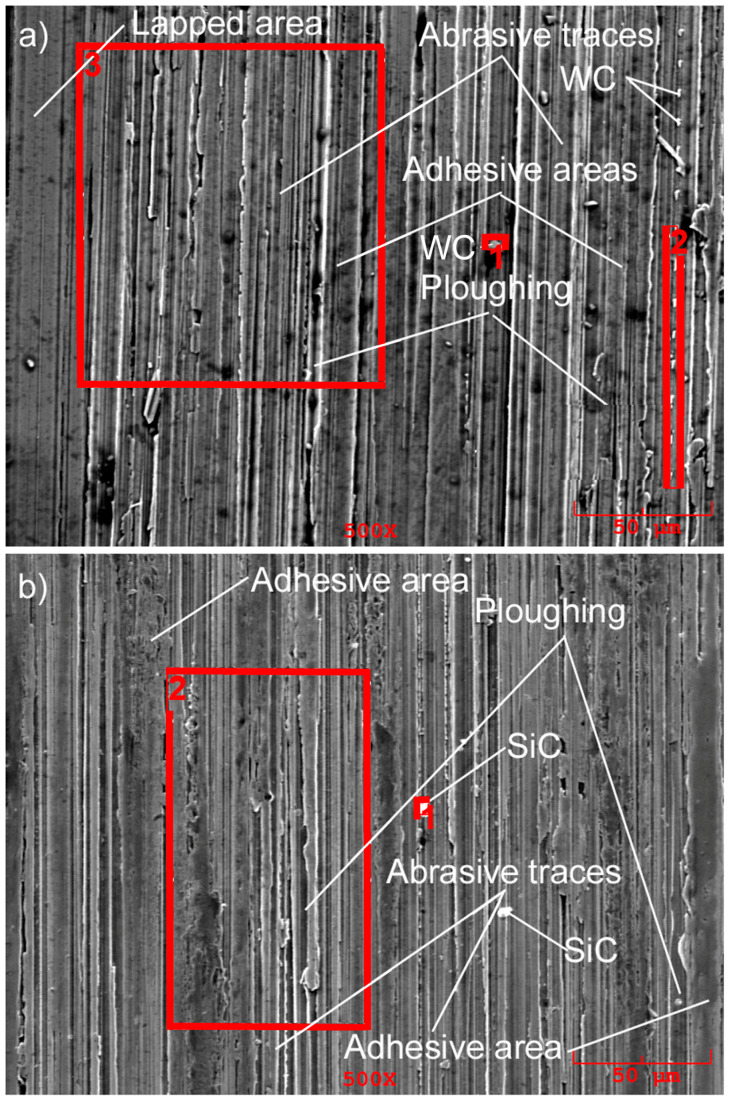
The morphology of the worn surfaces of counter-bodies formed after interaction with gray cast iron samples alloyed with WC (**a**) and SiC (**b**) carbides.

The morphology of worn surfaces on counter-bodies (rollers) is similar to the morphology of gray cast iron samples after laser alloying. Areas of abrasion and adhesion wear, lapped surfaces, and ploughing traces have been observed (Figure 15).

It is known in the literature that friction coefficients are one of the main factors in the characterization of tribologic properties [25,26]. The changes in the dry sliding friction of a modified aluminum bronze coating were studied by Yin et al. [4]. Moreover, Zhang et al. [27] analyzed the MFC evolution for the aluminum bronze coating; it was discovered that the MFC increased rapidly at the beginning of the test and then fluctuated greatly.

In this research, the effect of the laser hardening of gray cast iron is described. GJL 200 cast iron was used as the initial material, and the “Cometa” CO_2_ continuous-action laser was applied for the laser processing. The influence of laser processing has been investigated with laser beam diameters of 1−2 mm and laser beam speeds of 100−600 mm/min. The ground surface after laser alloying is more homogeneous, with a lower level of micro-roughness when compared to the initial cast iron. The technological parameters of laser alloying only marginally affect the parameters of surface roughness. Laser alloying of cast iron with tungsten and silicon carbides significantly changes the microstructure of the surface layer. High rates of heating and cooling of the surface layer melted by laser radiation lead to incomplete diffusion processes, the formation of a large number of different phases, and the formation of a fine-grained non-equilibrium structure. The speed of the laser beam profoundly influences the character of structural transformations both in the melting zone and in the heat-affected zones. The diameter of the laser beam is significantly less influential.

An important role in the changes in CoF, friction temperature, and wear intensity is played by the energy density of the laser spot. According to Campanelli et al. [28] surface energy density is treated as the combination of power, scan speed, and laser beam diameter. Moreover, similar relationships replacing the laser beam diameter with scanning hatch spacing were defined by Bai et al. [29]. The correlation between relative density, ultimate tensile strength, hardness, and surface roughness was also studied by Casalino et al. [30]. The main conclusion was that mechanical properties increase with increasing surface energy density, as the relative density follows the same relationship. However, the relationship between laser processing parameters and the wear rate is not clear. It depends not only on the energy density as a summative parameter but also on the complex phase-structural transformations and the formation of complex multicomponent compounds based on iron, carbon, tungsten, and silicon, the presence of which was confirmed in Section 3.6. Taking also into account the complex changes in heat fields as well as the destruction of the generated tribological film due to high mechanical loads, it can be concluded that ensuring a minimum wear rate of laser alloy coatings can be achieved by a synergistic adjustment of geometric, kinematic, and load factors.

Most relevant in practice is the consideration that when laser alloying with carbide particulates is applied to reinforced cast irons, the correct choice of the surface energy density ensures low, stable COFs and a considerable increase in wear resistance compared to cast iron in the input conditions. In the next stage of our research, the energy density of the laser spot will also be analyzed, as indicated in [4].

## 4. Conclusions

In this research, the following was revealed based on the research results:

- When gray cast iron is laser remelted with a layer of pre-coated carbides, a number of complex chemical compounds are formed, particularly various iron oxides, various iron-tungsten intermetallides, complex iron-tungsten carbides, W_2_C carbide, and a small amount of pure tungsten. When alloying cast iron with silicon carbides, various iron oxides, a number of iron silicides, and various modifications of silicon carbides are formed. Depending on the conditions of laser alloying, the amount of initial α–Fe changes, and austenite γ–Fe occurs.
- Depending on the laser processing parameters, the microhardness of cast iron after alloying with tungsten carbides increases 3.5–4.5 times, and after alloying with silicon carbides, it increases 3.0–4.0 times compared with the initial microhardness. The maximal microhardness was about 1050–1200 HV_100_ under WC alloying and about 750–1300 HV_100_ under SiC alloying, depending on laser power density. The thickness of the hardened alloyed layer with tungsten and silicon carbides, as well as the thickness of the HAZ, depends on the laser processing parameters, primarily on the laser beam speed.
- Laser processing conditions, alloying material, and time of friction have no effect on the momentary COFs of cast iron after laser alloying samples. A double loading increase decreases the COF by 1.2 times due to changes in the character of friction surfaces’ wear. The temperature in the friction zone stays practically constant regardless of the alloying particulates and laser processing parameters.
- The correct choice of laser alloying conditions allows the wear resistance of cast iron to be increased by 4–24 times after the addition of WC in comparison with the initial material and after the addition of SiC, where it then increases by 2–18 times. Under the influence of the high density of surface energy on the worn surface, there are areas where the mutual overlapping of the sample and counter-body materials takes place and, as a consequence, the friction coefficient decreases. Small particles of WC and SiC carbides dissolve in the cast iron matrix during laser remelting, while larger ones remain in the surface layer, activating abrasive wear and causing the ploughing process. A tribofilm is formed on the friction surfaces, reducing and stabilizing the COF and increasing wear resistance.

## Figures and Tables

**Table 1:** Codes and values of the factors used in the studies.

The Test Number	Laser Spot Diameter		Laser Spot Speed		Surface Energy Density, J/mm^3^
	Code X_1_	Value, mm	Code X_2_	Value, mm/min	
1	−1	1	−1	100	765
2	+1	2	−1	100	382
3	−1	1	+1	600	128
4	+1	2	+1	600	64

**Table 2:** Typical chemical composition of the worn surfaces of gray cast iron samples after laser alloying with WC carbides.

Area Nr.	Chemical Elements, %				
	Fe	C	W	P	S
1	86.7	10.02	2.46	0.24	0.58
2	78.33	17.75	3.02	0.25	0.65

**Table 3:** Typical chemical composition of the worn surfaces of gray cast iron samples after laser alloying with SiC carbides.

Area Nr.	Chemical Elements, %				
	Fe	C	Si	P	S
1	81.7	15.53	1.86	0.17	0.75
2	94.7	2.91	1.94	0.15	0.29

**Table 4:** Typical chemical composition of the worn surfaces of counter-bodies formed after interaction with gray cast iron samples alloyed with WC carbides.

Area Nr.	Chemical Elements, %				
	Fe	C	W	P	S
1	62.66	35.87	1.19	0.16	0.12
2	91.28	7.09	1.38	0.11	0.14
3	88.92	9.44	1.21	0.21	0.22

**Table 5:** Typical chemical composition of the worn surfaces of counter-bodies formed after interaction with gray cast iron samples alloyed with SiC carbides.

Area Nr.	Chemical Elements, %				
	Fe	C	Si	P	S
1	61.44	38.11	0.16	0.08	0.21
2	93.44	5.1	0.16	0.76	0.72

## Data Availability

Not applicable.
